# Whole-Genome Sequences of Zika Virus FLR Strains after Passage in Vero or C6/36 Cells

**DOI:** 10.1128/genomeA.01528-17

**Published:** 2018-01-25

**Authors:** Lindsey A. Moser, Lauren M. Oldfield, Nadia Fedorova, Vinita Puri, Susmita Shrivastava, Paolo Amedeo, Richard Isom, Lihui Hu, Alan Durbin, Iara Rocchi, Torrey Williams, Gene S. Tan, Reed S. Shabman, Kristen A. Bernard, Brett E. Pickett

**Affiliations:** a University of Wisconsin–Madison, Madison, Wisconsin, USA; b J. Craig Venter Institute, Rockville, Maryland, USA; c J. Craig Venter Institute, La Jolla, California, USA

## Abstract

We report 26 complete genomes of Zika virus (ZIKV) isolated after passaging the Zika virus strain FLR in mosquito (C6/36) and mammalian (Vero) cell lines. The consensus ZIKV genomes we recovered show greater than 99% nucleotide identify with each other and with the FLR strain used as input.

## GENOME ANNOUNCEMENT

Zika virus (ZIKV) belongs to the *Flavivirus* genus within the *Flaviviridae* family and has a single-stranded positive-sense RNA genome that is approximately 10.8 kb in length. Like many flaviviruses, ZIKV is an arbovirus, cycling between mosquito and mammalian hosts (1). Here, we report the whole-genome sequences for parental and biologically cloned ZIKV FLR strains derived from C6/36 mosquito or Vero mammalian cells that displayed tiny, small, medium, or large plaque phenotypes on Vero cells.

The ZIKV FLR strain was originally isolated from the blood of a human in Barranquilla, Colombia, in December 2015 and was passaged twice on C6/36 cells prior to BEI deposit and a third time at BEI Resources (NR-50183) (2). For this study, parental ZIKV FLR stock virus was first passaged on Vero (ATCC CCL-81) or C6/36 (ATCC CRL-1660) cells. Subsequently, biological clones were isolated by three rounds of selection for different-sized plaques with amplification on either C6/36 or Vero cells. The parental Vero cell-derived ZIKV FLR virus was passaged once in Vero cells prior to sequencing (MF574552). The Vero cell-derived isolates, biological clones of the parental virus with three additional passages in Vero cells, have been deposited in GenBank under the following accession numbers: MF574555, MF574557 to MF574562, MF574565, MF574567, MF574568, MF574570 to MF574572, MF574575, and MF574576. The parental C6/36 cell-derived ZIKV FLR was passaged once in C6/36 cells prior to sequencing (MF574553). The C6/36 cell-derived isolates, biological clones of the parental virus with three additional passages in C6/36 cells, have been deposited in GenBank under the following accession numbers: MF574554, MF574556, MF574563, MF574564, MF574566, MF574569, MF574573, MF574574, and MF574577.

Viral RNA was extracted from the supernatant of each cell culture and then subjected to reverse transcription with random hexamers or nonamers prior to cDNA-based or PCR amplicon-based sequence-independent single-primer amplification (SISPA), as described previously (3, 4). Next-generation sequencing libraries were then constructed, and the samples were sequenced on the Ion Torrent PGM or Illumina MiSeq platform (2 × 300 bp). This approach enabled the unbiased identification of high-quality nucleotide substitutions that occurred at the consensus level for each passaged strain.

Sequencing reads were demultiplexed by barcode, trimmed, and then *de novo* assembled. BLASTN searches of these contigs were performed against the GenBank nonredundant nucleotide database (5). Following *de novo* assembly, raw reads were mapped to the most appropriate ZIKV reference genome using a reference-based assembly in the CLC bio software suite. In total, over 233,000 reads were assembled for this collection of viruses (mean, 9,168; median, 8,467; range, 1,302 to 13,643). The mean nucleotide identity across strains was calculated to be 99.999% (range 99.995% to 100%).

In summary, we identified very few differences between the various ZIKV FLR strains at the consensus level, despite the different plaque phenotypes that were observed. These sequence data can increase our understanding of variations that may contribute to host specificity.

### Accession number(s).

The consensus sequences for these viruses were validated manually and annotated with VIGOR (6) prior to GenBank submission. These sequences were assigned GenBank accession numbers MF574552 through MF574577 under BioProject identification number PRJNA314889.
